# Early rigorous control interventions can largely reduce dengue outbreak magnitude: experience from Chaozhou, China

**DOI:** 10.1186/s12889-017-4616-x

**Published:** 2017-08-02

**Authors:** Tao Liu, Guanghu Zhu, Jianfeng He, Tie Song, Meng Zhang, Hualiang Lin, Jianpeng Xiao, Weilin Zeng, Xing Li, Zhihao Li, Runsheng Xie, Haojie Zhong, Xiaocheng Wu, Wenbiao Hu, Yonghui Zhang, Wenjun Ma

**Affiliations:** 1 Guangdong Provincial Institute of Public Health, Guangdong Provincial Center for Disease Control and Prevention, No. 160, Qunxian Road, Panyu District, Guangzhou, 511430 China; 2 Guangdong Provincial Center for Disease Control and Prevention, No. 160, Qunxian Road, Panyu District, Guangzhou, 511430 China; 3 Faculty of Medical Statistics and Epidemiology, School of Public Health, Sun Yat-sen University, Guangzhou, 510080 China; 4 School of Public Health and Social Work & Institute of Health and Biomedical Innovation, Queensland University of Technology, QLD, Brisbane, Australia

**Keywords:** Dengue fever, Epidemic, Compartmental dynamic model, SEIR model

## Abstract

**Background:**

Dengue fever is a severe public heath challenge in south China. A dengue outbreak was reported in Chaozhou city, China in 2015. Intensified interventions were implemented by the government to control the epidemic. However, it is still unknown the degree to which intensified control measures reduced the size of the epidemics, and when should such measures be initiated to reduce the risk of large dengue outbreaks developing?

**Methods:**

We selected Xiangqiao district as study setting because the majority of the indigenous cases (90.6%) in Chaozhou city were from this district. The numbers of daily indigenous dengue cases in 2015 were collected through the national infectious diseases and vectors surveillance system, and daily Breteau Index (BI) data were reported by local public health department. We used a compartmental dynamic SEIR (Susceptible, Exposed, Infected and Removed) model to assess the effectiveness of control interventions, and evaluate the control effect of intervention timing on dengue epidemic.

**Results:**

A total of 1250 indigenous dengue cases was reported from Xiangqiao district. The results of SEIR modeling using BI as an indicator of actual control interventions showed a total of 1255 dengue cases, which is close to the reported number (*n* = 1250). The size and duration of the outbreak were highly sensitive to the intensity and timing of interventions. The more rigorous and earlier the control interventions implemented, the more effective it yielded. Even if the interventions were initiated several weeks after the onset of the dengue outbreak, the interventions were shown to greatly impact the prevalence and duration of dengue outbreak.

**Conclusions:**

This study suggests that early implementation of rigorous dengue interventions can effectively reduce the epidemic size and shorten the epidemic duration.

## Background

Dengue fever is a mosquito-borne viral disease with extensive global distribution; half the world’s population is at risk of infection [1]. China has experienced a rapid increase in the number of dengue cases over the past two decades [2]. Guangdong province in the south of China has experienced the highest burden [3, 4]. In 2014, this province witnessed a large dengue outbreak with a total of 45,224 cases and six deaths; cases were recorded in 20 of 21 prefectures [2, 5, 6].

In August of 2015, an unprecedented outbreak of serotype 2 dengue virus occurred in Chaozhou city of Guangdong Province. The first indigenous case was reported on August 19^th^. Daily case numbers peaked on 13^th^ of September (90 cases) and by the end of 2015 a total of 1380 cases had been recorded. Initially, the local government initiated routine control measures. These routine control measures, enacted by public health professionals from the local Center for Disease Control and Prevention (CDC), mainly included control of mosquitos using space spraying and fogging of pesticides in outdoor areas around (within 200 m) the residence of notified cases. By September 8, however, the total number of cases had increased to 95, forcing authorities to implement more rigorous control measures which required the participation of other departments and the community. These measures included: (1) enhancing leadership by establishing a dengue control committee with members from various departments including: the health administrative department, disease control and prevention center, hospitals, education department, street governments and communities; (2) conducting daily risk assessments to adjust control measures based on adult mosquito density and Breteau Index; (3) increased mosquito and larvae eradication in high risks areas. Professional workers from CDC system went to the communities to inspect and remove all potential egg laying sites door to door. Health education professionals taught people the basic information about dengue fever and mosquito, and how to protect themselves from mosquito biting, such as using insect repellants, and keeping windows, doors and porches tighly screened. All community members were motivated to remove any sources of standing water and aquatic plants, and keep weeds and other vegetation mowed to minimize the shelter for adult mosquitoes. Larvacides and pesticides were sprayed and fogged by special equipment to effectively kill the mosquito larvae and adult mosquitos. Pesticide products, such as spray liquid and mosquito-repellent incenses, were distributed to very family for daily mosquito eradication; (4) finally, hospitals were required to accelerate the process of dengue case diagnosis, reporting and treatment.

These intensified efforts were successful in controlling the outbreak. However, some questions arising from this event need to be answered to better guide dengue outbreak management and control in the future. It is currently unknown the degree to which intensified control measures reduced the size of the epidemics, and when, in future, should such measures be initiated to reduce the risk of large dengue outbreaks developing?

Mathematical models have been widely used to understand various driving factors of dengue infection and transmission [1, 2]. Such models usually pay special attention to the transmission dynamics between hosts, vectors, and dengue virus. Here we employed a mathematical SEIR (Susceptible, Exposed, Infected and Removed) model to answer the above questions [7, 8]. SEIR model is a compartmental dynamic system that simulates the evolution of incidence rate under specific conditions. This model divides the total human population (Nh) into four groups [9]: people who are susceptible to being infected with dengue virus (S_h_), people who have been exposed to the virus (E_h_), people who are infected (I_h_) and people who have recovered (R_h_). Adult female mosquitoes (N_v_) can also be categorized as those that are susceptible with dengue virus (S_v_), mosquitoes that have been exposed to the virus (E_v_), and mosquitoes that are infected with dengue virus (I_v_). Humans enter the susceptible class through birth, and become infected with a finite probability after being fed upon by an infectious mosquito. After being successfully infected, humans move from the susceptible class (S_h_) to the exposed class (E_h_), and, after an intrinsic incubation period, they move to the infectious class, (I_h_). Finally after a period of time, these humans recover and move to the recovered class R_h_. For the purposes of modelling, it is assumed that recovered humans have immunity to dengue for life. SEIR models were developed and have been employed widely throughout the world to assess the effectiveness of control measures for infectious diseases [7, 8]. SEIR models, however, have not been extensively used to simulate the dengue transmission in China, the region with high dengue incidence rate especially in Guangdong province [3]. The application of SEIR modelling to China could provide information integral to the development of more effective dengue control measures.

## Study setting

**Fig. 1:**
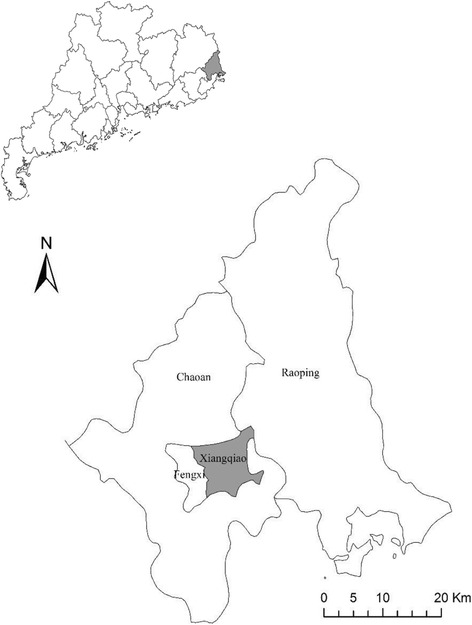
The location of Xiangqiao district in the city of Chaozhou, Guangdong province, China. Note: This figure depicts the distribution of Xiangqiao district in Chaozhou city through ArcGis (ArcMap 9.3, Environmental Systems Research Institute, Redlands, USA)

Chaozhou, located in the east of Guangdong Province (Fig. 1), covers an area of 3613.4 km^2^ and is inhabited by 2.67 million people. The city exhibits a subtropical monsoonal climate with distinct seasons and abundant rainfall. Chaozhou is colloquially known as the “Chinese Porcelain City” owing to its industries that produce and export ceramic globally. The abandoned ceramics could provide abundant of breeding places for mosquitos after raining. Chaozhou is also referred to as the “Famous Hometown of Overseas Chinese”, owing to the thousands of people who emigrated abroad. A large number of these expatriates return on a year basis to visit friends and family. The high degree of connectedness of Chaozhou with the rest of the world makes the city vulnerable to the importation of infectious diseases, such as dengue. Further adding to this risk is the local tradition of planting flowers, particularly aquatic plants, at home or on the roof of houses providing breeding environment for mosquito vectors such as *Aedes albopictus*, the major vector of dengue virus in Chaozhou [10]. Xiangqiao district, located in the center of Chaozhou city, has the highest density of population among all districts (Fig. 1). In this dengue outbreak, the majority of the indigenous cases (90.6%) were reported in Xiangqiao district. Therefore, we conducted all SEIR model analyses in the Xiangqiao district, and all the parameters were set according to the situations of this district. We aimed to evaluate the effectiveness of different control levels and timing of starting the control measures on the epidemic dynamics and pattern of dengue outbreak.

## Methods

Daily dengue case numbers were collected for the period of August 19^th^ to December 31^st^, and Breteau Index (BI, an index used to evaluate the number of containers positive for Aedes larvae or pupae per 100 houses [11]) was determined from September 2^nd^ to October 28^th^ in Xiangqiao district, Chaozhou city. All data on dengue cases and BI were obtained from Guangdong Provincial Center for Disease Control and Prevention that has the access to the National Selected Infectious Diseases and Vectors Surveillance System. The BI data were daily reported by public health professionals in monitoring sites located in most streets and towns to the Guangdong Provincial Center for Disease Control and Prevention. ArcGis (ArcMap 9.3, Environmental Systems Research Institute, Redlands, USA) was used to describe the locations of Chaozhou city and its districts. Then we used a SEIR model to simulate the daily dengue cases under different control scenarios [7]. The SEIR model can be written as follows:

1 $$ \frac{dS_h}{dt}={\varPsi}_h{N}_h-{\beta}_{vh}\frac{I_v}{N_h}{S}_h-{\mu}_h{S}_h $$

2 $$ \frac{dE_h}{dt}={\beta}_{vh}\frac{I_v}{N_h}{S}_h-\left({\upsilon}_h+{\mu}_h\right){E}_h $$

3 $$ \frac{dI_h}{dt}={\upsilon}_h{E}_h-\left({\gamma}_h+{\mu}_h\right){I}_h $$

4 $$ \frac{dR_h}{dt}={\gamma}_h{I}_h-{\mu}_h{R}_h $$

5 $$ \frac{dS_{\nu }}{dt}={r}_v\left(1-\frac{N_v}{K}\right){N}_v-{\beta}_{h\nu}\frac{I_h}{N_h}{S}_{\nu }-\left({\mu}_{\nu }+\delta \right){S}_{\nu } $$

6 $$ \frac{dE_{\nu }}{dt}={\beta}_{hv}\frac{I_h}{N_h}{S}_{\nu }-\left({\nu}_{\nu }+{\mu}_{\nu }+\delta \right){E}_{\nu } $$

7 $$ \frac{dI_{\nu }}{dt}={\nu}_{\nu }{E}_{\nu }-\left({\mu}_{\nu }+\delta \right){I}_{\nu } $$

The state variables for the Eqs. (1–7) are shown in Table 1.

**Table 1:** State variables for the SEIR equation models

Variables	Explanation	Values	Sources
S_h_	Number of susceptible humans	-	-
E_h_	Number of exposed humans	-	-
I_h_	Number of infectious humans	-	-
R_h_	Number of recovered humans	-	-
S_v_	Number of susceptible mosquitoes	-	-
E_v_	Number of exposed mosquitoes	-	-
I_v_	Number of infectious mosquitoes	-	-
N_h_	Total human population size	600,000	statistic yearbook
N_v_	Total mosquito population size	-	-
Ψ_h_	Per capita birth rate of humans. Time^−1^	1/(70*360)	Estimation
S_v_(0)	Initial values of susceptible mosquitoes	1,987,800	MCMC Fitting
E_v_(0)	Initial values of exposed mosquitoes	434	MCMC Fitting
I_v_(0)	Initial values of infectious mosquitoes	20	MCMC Fitting
*δ*	Reduction rate of mosquitoes due to control measures. Time^_1^	varied	MCMC Fitting
β_hv_	Effective contact rate from an infected human to a susceptible mosquito. Dimensionless	0.576	MCMC Fitting
β_vh_	Effective contact rate from an infected mosquito to a susceptible human. Dimensionless	0.426	MCMC Fitting
ν_h_	Per capita rate of progression of humans from the exposed state to the infectious state. Time^_1^	1/5	[13]
ν_v_	Per capita rate of progression of mosquitoes from the exposed state to the infectious state. Time^_1^	1/10	[13]
γ_h_	Per capita recovery rate for humans from the infectious state to the recovered state. Time^_1^	1/6	[13]
μ_h_	Per capita death (and emigration) rate for humans. Time^_1^	1/(70*360)	Estimation
μ_v_	Density-independent death rate for mosquitoes. Time^_1^	1/21	[13]
K_v_	Carrying capacity of mosquitoes. Mosquitoes	28,200	MCMC Fitting
r_v_	Natural growth rate of mosquitoes. Time^_1^	0.013	MCMC Fitting
A	Scale parameter	0.373	MCMC Fitting

-: The parameters were described in the methods section

We defined N_h_ as the total population in Xiangqiao district (Ns = 0.6 million). Some undetermined parameters (e.g., the initial values of S_v_, E_v_ and I_v_, the transmission rate and growth rate of mosquitoes) were fitted by Markov chain Monte Carlo (MCMC) method [12]. Other parameters were referred to related studies [13].

The local government and the public health agencies in Chaozhou had taken various measures to control the mosquitoes. To assess the effects of control intensities on the epidemic, we inserted a reduction rate *δ* into the dynamic equations of mosquitoes (e)-(g). We used the cumulative daily BI as an alternative indicator to reflect the dynamic change in mosquito density. The formula is written as follows:

$$ \delta (t)=-A \ln \left(\frac{\sum_{i=1}^{\tau}\left(B(t)+\cdots +B\left(t+i\right)\right)}{\sum_{i=1}^{\tau}\left(B\left(t-1\right)+\cdots +B\left(t-i\right)\right)}\right), $$

Where *A* is a scale parameter and *B*(*t*) is the BI value in day t. The above equation means that the variation of mosquito number due to control at one time step, i.e., *N*_*v*_(*t*)/*N*_*v*_(*t* − 1) = exp(−*δ*), is proportion the ratio of cumulative BI values before *τ* days to those after *τ* days. The sale constant *A* will be estimated by MCMC method. Here we assume*τ* = 3.

Parameters were estimated by sampling the posterior distribution, using the Metropolis-Hasting MCMC algorithm [12]. Nine chains with initial conditions were explored to examine the convergence of posterior distribution. The prior distributions of the parameters follow Gaussian with a mean of zero. After a number of iterations, we can then analyze the statistics of the model parameters and estimate their values. Using these parameter in model (e)-(g), we explored the time evolution of model variables.

Since the common used intervention measures of dengue control were controlling mosquitoes or protecting people against their bites, these methods would be simulated in our model by reducing the density of female mosquitoes and reducing the effective contact rate (which is equal to the product of infection probability by one contact and contact frequency at unit time).

To evaluate the effects of different control strategies, we substituted various effective contact rate and mosquito reduction rate into the model (e)-(g). For example, without control means *δ* = 0; 5% daily reduction of mosquito density at day *t* means *δ*(*t*) =  − log 0.95, and conducting control intervention during a period means that the parameter *δ* is equal to certain positive value during this period and is equal to zero at other time. We first estimated the incidence rate without control by running the model with *δ* = 0, and then reduced 5%, 10%, and 15% of the effective contact rate to estimate the role of this rate in dengue transmission. We further supposed 5%, 10%, and 15% reduction of the mosquito density to understand the outcome of such control measures. In view of the limited control resources in some regions, we finally estimated the effects of intervention between a short period and every few days. The results were presented in the next section.

All statistical analyses were conducted in Matlab (R2016a).

## Results

**Fig. 2:**
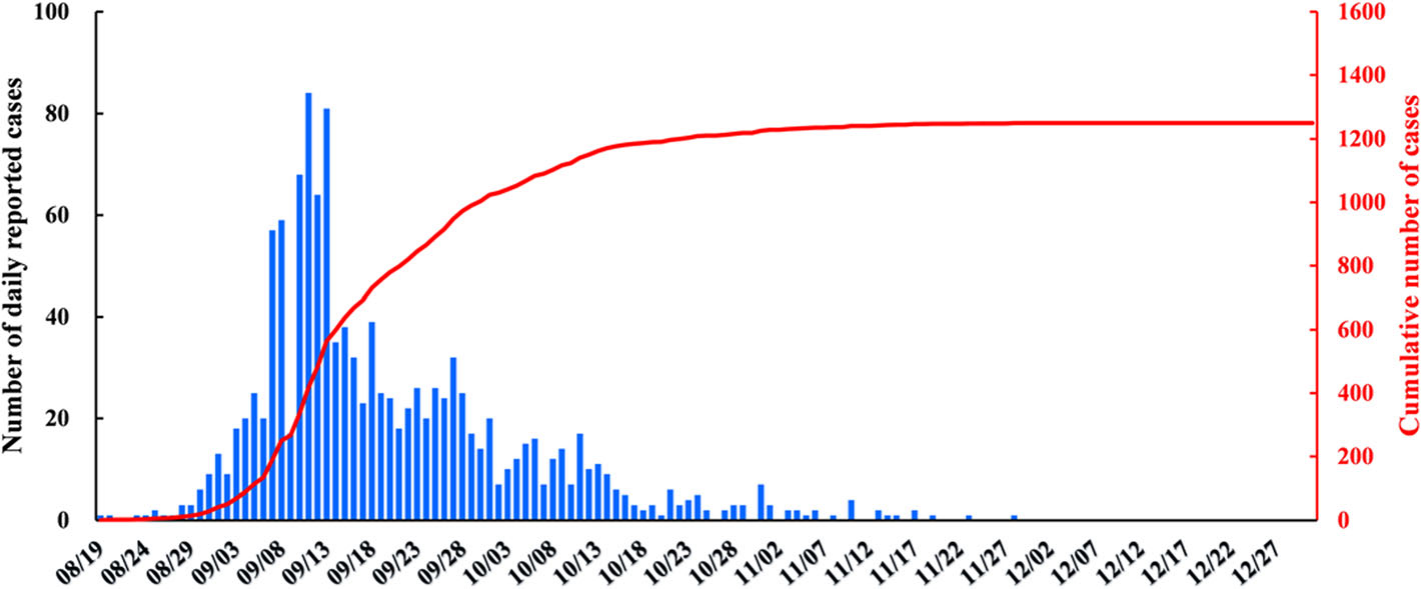
The temporal distribution of all reported local dengue cases in Xiangqiao district, Chaozhou city, China

**Fig. 3:**
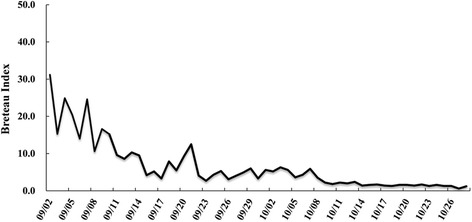
Daily Breteau Index during the dengue outbreak in Xiangqiao district, Chaozhou, China

A total of 1250 indigenous dengue cases were reported in Xiangqiao district, Chaozhou from August 19^th^ to December 31^st^ 2015. Of these 659 (52.7%) were females, and 359 (28.7%) were elderly (≥60 years) (Table 2). The first indigenous case was reported on August 19^th^, after which the daily number of cases rapidly increased to a daily peak (*n* = 84) on September 11^th^, and then the epidemic rapidly declined to around 20 cases per day within two weeks. Within the days after September 31^st^, the number of daily cases was gradually reduced to a very low level (Fig. 2). The BI gradually declined from 31.2, prior to September 2^nd^ to lower than 5.0 after October 10^th^ (Fig. 3).

**Table 2:** General characteristics of local dengue cases in Xiangqiao district, Chaozhou, China

	Number of cases N (%)
Gender	
Males	591 (47.3)
Females	659 (52.7)
Age (years)	
< 20	124 (9.9)
20~	157 (12.6)
30~	168 (13.4)
40~	183 (14.6)
50~	259 (20.7)
60~	240 (19.2)
≥ 70	119 (9.5)

We first evaluated the effectiveness of actual control interventions using BI as an indicator. By integrating it into the transmission model, and using the MCMC method, we estimated the unknown model parameters. Their estimated values are showed in Table 1. Running the model with these parameters, we obtain a series of results as follows.

**Fig. 4:**
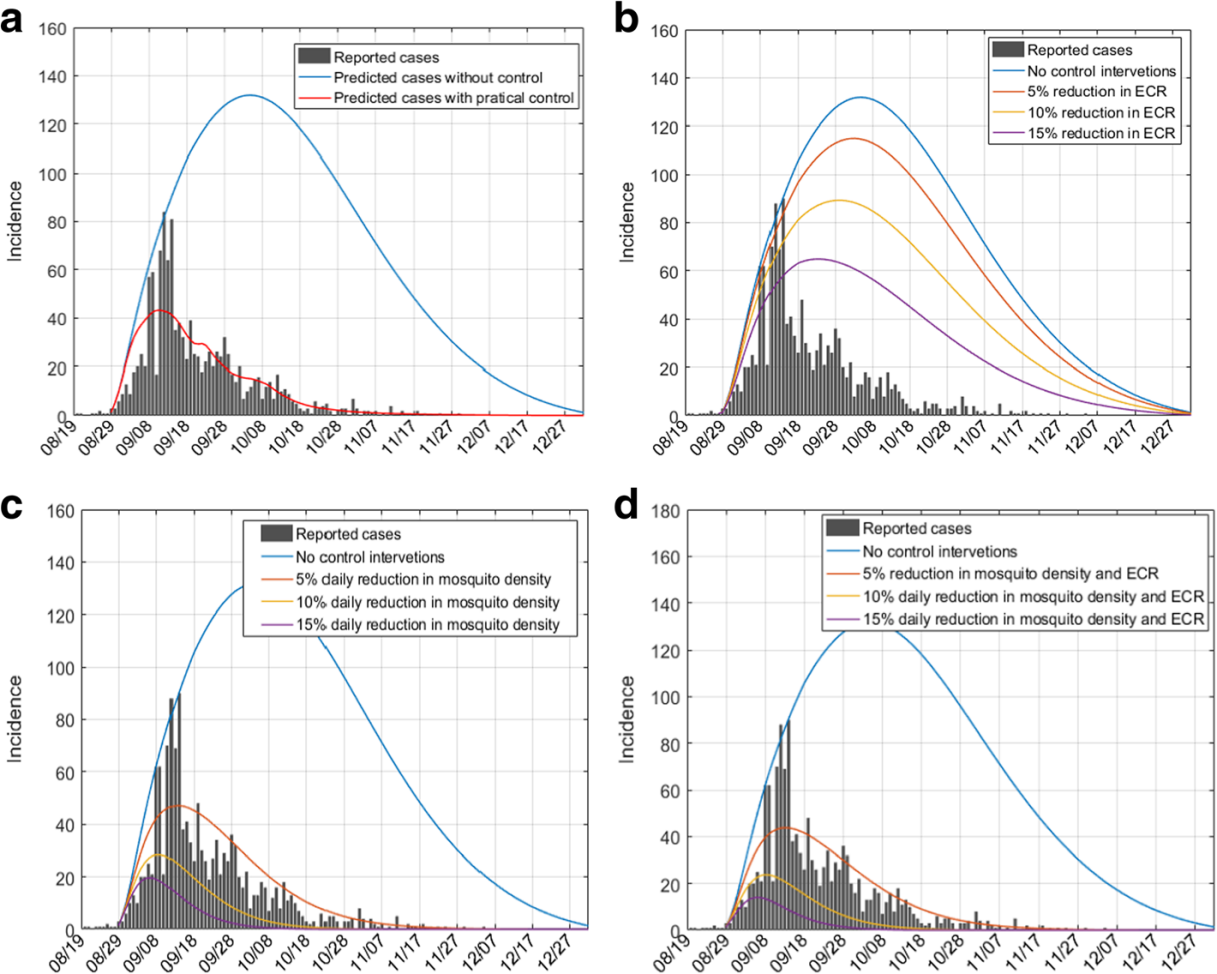
Effects of different levels of control interventions on dengue epidemics in Xiangqiao district, Chaozhou, China. Chart **a**: Comparisons of the effect of no control and practical control interventions on dengue epidemics.Chart **b**: Effects of different reductions in effective contact rate (ECR) on dengue epidemics.Chart **c**: Effects of different reductions in mosquito density on dengue epidemics.Chart **d**: Effects of different reductions in both mosquito density and ECR on dengue epidemics

The modeling results showed a total of 1255 dengue cases, that is close to the reported number (*n* = 1250), and their correlation coefficients is 0.88 (*p* < 0.001). Meanwhile, the simulated number of daily cases was less than 1 after November 8^th^ 2015 (Fig. 4a).

Figure 4 shows the modeling results in case of different levels of control interventions. It is observed in Fig. 4a that if there were no control interventions, there would have been more than 8200 indigenous dengue cases with a peak of 132 daily cases in October 3^th^, and transmission would have continued until the end of December. Furthermore, Fig. 4b-d indicates that reducing effective contact rate and especially reducing mosquito density could dramatically reduce the epidemic magnitude and shorten the epidemic period. For instance, the total number of indigenous cases would decline from 8200 to 5328 and 655, if the effective contact rate was reduced by 10% and mosquito density was reduced by 5% everyday, respectively. The epidemic period would be correspondingly shortened. For example, the daily number of cases would be less than one in the middle of October if the mosquito density was daily reduced by 5%. Similar effects on the epidemic pattern can be found when reducing effective contact rate. In addition, if transmission rate and mosquito density were reduced simultaneously, the effect was magnified. For example, the total number of cases was 529 if the transmission rate and mosquito density were simultaneously reduced by 10%. We found that reducing mosquito density is more effective than reducing effective contact rate, which implied that efficient control of mosquitoes is crucial in dengue surveillance.

**Fig. 5:**
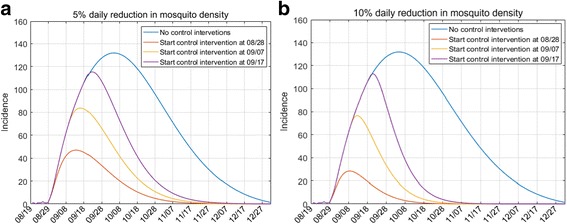
Effects of start timing for control interventions on dengue epidemic outbreaks. Chart **a**: The mosquito density was daily reduced by 5% caused by the control interventions. Chart **b**: The mosquito density was daily reduced by 10% caused by the control interventions

Next, we assessed the effects of start timing of control interventions after dengue outbreak. The results shown in Fig. 5 indicate that the effects of interventions are more pronounced if they were carried out earlier, and the control effect was still significant even after one month of the outbreak. For example, if the interventions, including daily reducing 5% and 10% of mosquito density, had been implemented on the 9^th^ day after the first infection, the total number of dengue cases would have been restricted to 1645 and 655, where 6588 and 7578 cases could be avoided compared with no control scenario, respectively. Even if the control (daily reducing 10% of mosquito density) was initiated at the 30^th^ after the first infection, more than 2800 dengue cases would have been prevented and the outbreak would be ended earlier compared with no control scenario. The results showed that early intervention of killing mosquitoes would be more effective for dengue control. Such intervention caused the epidemic terminating earlier, and the more intensive of mosquito control, the shorter of the epidemic.

**Fig. 6:**
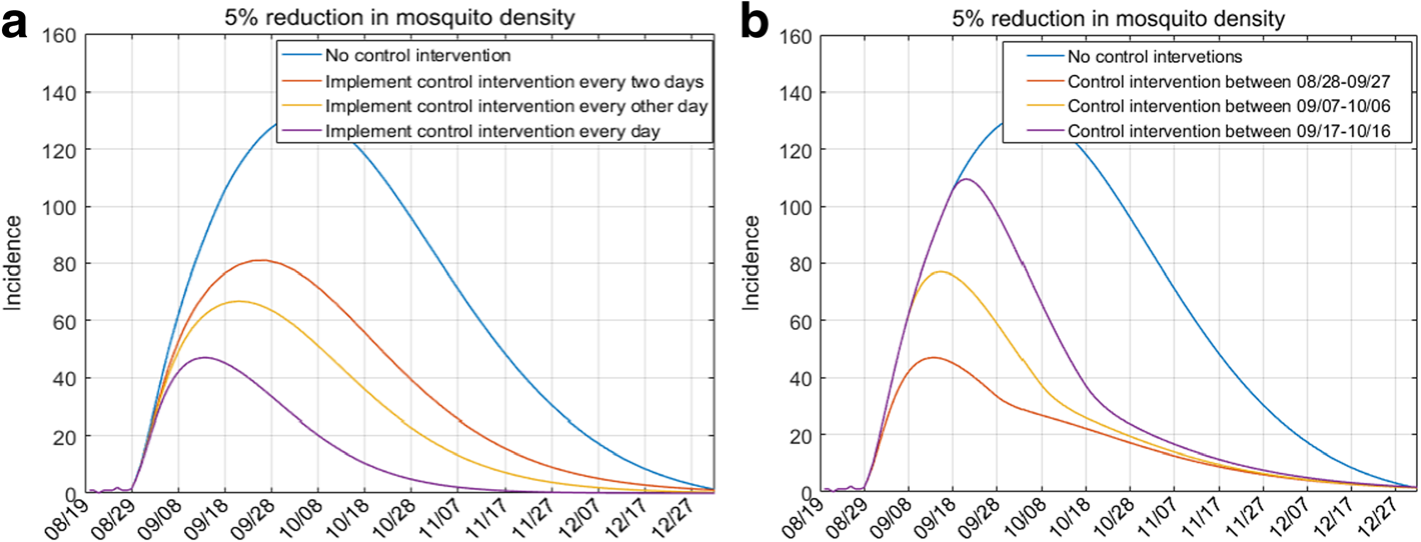
Effects of time interval for control interventions on dengue epidemic outbreaks. 5% reduction in mosquito density means that the control intervention causes the mosquito density reducing 5% compared to the day before. Chart **a** The control interventions were assumed to be carried out from August 28^th^. Chart **b** The control interventions were assumed to be carried out between 08/28–09/27, 09/07–10/06 and 09/17–10/16, respectively

In the following, we evaluated the effects of time interval for control interventions, in the light of that the control interventions may be just persisted for a while due to the limited financial and human resources. As shown in Fig. 6, we observed that impulse mosquito control is also effective for dengue control. In particular, if the control interventions were carried out every two days and every other day, 3825 and 5026 cases could be avoided during the epidemic, respectively. Furthermore, control interventions implemented between August 28^th^ and September 27^th^, and between September 7^th^ and November 6^th^, could avoid 5958 and 5011 cases, respectively. These above results of control effects are compared with the scenario of no control interventions. These results indicate that implementing intervention only for 30 days can also reduce a large proportion of dengue cases, and the sooner the better, but the epidemic would persist at a low level. The results also indicate that continuous intervention is better for dengue control.

## Discussion

In this study, modelling approach indicated that the practical control interventions against mosquitos can significantly decrease the number of dengue cases and shorten the epidemic duration in Chaozhou, China. Some other countries also experienced similar process as we did [14, 15]. For example, during the dengue outbreak in Cuba in 1981, the government successfully controlled the epidemic by implementing a rigorous vector control program based on intensive insecticidal treatment followed by reduction of available larval habitats [14]. Singapore also successfully prevented dengue using vector control strategies, in which public education and law enforcement are two key elements [15]. This provides evidence that comprehensive, continuous and intensified mosquito control interventions are effective in response to dengue outbreak.

Our modelling results further demonstrated that rigorous and comprehensive interventions are more effective compared with routine interventions. If the effective contact rate and mosquito density decreased simultaneously, the synergetic effects of these strategies were much stronger than their individual effects. Luz et al. also found that one or more applications of high-efficacy larval and adult vector control could significantly decrease the dengue burden for up to 2 to 4 years, but the intermediate or low efficacy control had less effect [16]. A recent study conducted by Tang et al. which focused on the 2014 dengue outbreak in Guangzhou also demonstrate the similar results [17]. World Health Organization (WHO) recommended comprehensive control strategies in response to dengue epidemic including administrative, legislative, regulatory, legal and media advocacy as well as social mobilization [18]. This implied that a comprehensive rigorous control strategy is very important for the control and prevention of dengue epidemic outbreaks.

We also found that the control approaches to be more effective if they were implemented earlier. For instance, the number of cases could be reduced by more than 90% if rigorous interventions were initiated after 9 days of the dengue epidemic onset compared to without control scenario. This is consistent with several other studies [7, 17, 19, 20]. Karl et al. assessed the effect of variation of control measures on a dengue epidemic outbreak in Cairns, Australia in 2003. The authors observed that if the vector control was initiated 2 weeks earlier than occurred in the actual outbreak, the number of cases would have been reduced by 50%. By contrast, initiating intervention 2 weeks later than the original date approximately doubled the predicted median number of cases [21]. These findings demonstrate the imperativeness of initiating early interventions in controlling dengue epidemics. Our study also demonstrated that control measure is effective and necessary during the process of infection. Furthermore, we found that the intervention carried out for a short time or every one/two days still work for dengue control, but it should be carried out as soon as possible. It was observed that continuous intervention at the beginning seems to be more effective. This finding is important for dengue control in practice, especially for the regions where there is no adequate preparedness and resources.

## Strengths and limitations

This is the first study using a SEIR model to evaluate the effects of control interventions for a serotype 2 dengue outbreak in Chaozhou, China. In this study we simultaneously assessed the effectiveness of control measures on *Aedes albopictus* density and effective contact rate between mosquito and humans, and different timing of starting these measures. These results are profoundly significant for local decision makers and stakeholders in establishing policies and strategies to guide the dengue outbreak control in China. These finding also have implications for public health authorities in other countries.

There were also some limitations to this study. Firstly, mosquito parameters used in this SEIR model are assumed to be constant, which was not tenable in practice. In fact, the parameters (e.g., contact rate, growth rate, death rate, extrinsic incubation period) are affected by many factors, such as temperature, rainfall, human movement and exposure outside the residence [22]. The estimated values of these parameters are averaged in our model by using MCMC method. A stochastic model or deterministic model with time-varied parameters might provide more accurate information on dengue epidemic [23]. Secondly, BI was temporarily monitored by the local public health professionals during the outbreak. The number and locations of monitoring sites might be changed due to the changing of epidemic situation. New monitoring sites might be added in some places when dengue cases were reported for the first time, which might be an important reason leading to the instability of BI. For instance, a large fluctuation of BI was found during the September 20^th^ to 21^st^, which may reduce the representativeness of BI on the control intensity all over the city. Thirdly, asymptomatic dengue fever infection was not tested in the population, which might be an important reason for inconsistent results between the reported number and the modeled number. For example, fewer reported cases were observed in the early stage of epidemic compared with the simulated number, and the number of reported cases suddenly jumped to a very high value and then abruptly dropped down (Fig. 4a).

## Conclusions

In summary, the practical control interventions made significant effects on controlling the dengue epidemic outbreak in Chaozhou, China. Reduction of the effective contact rate especially the mosquito density could dramatically reduce the epidemic magnitude and shorten the epidemic period, and their synergetic effects could be magnified if they were reduced simultaneously. The effects of interventions were more pronounced if they were started earlier, and the control effect was still significant even after one month of the outbreak. Moreover, the continuous intervention could be better for dengue control, but if there is not enough resources and the interventions just can persist for a while, then it should be carried out as soon as possible after the outbreak. In summary, early and rigorous comprehensive control interventions are strongly recommended in the future dengue outbreaks.

## Abbreviations

BI: Breteau Index
E_h_: People who have been exposed to the virus
E_v_: Mosquitoes that have been exposed to the virus
I_h_: People who are infected
I_v_: Mosquitoes that are infected with dengue virus
MCMC: Markov chain Monte Carlo
N_h_: Total human population
N_v_: Total adult female mosquitoes
R_h_: People who have recovered
SEIR: Susceptible, Exposed, Infected and Removed model
S_h_: People who are susceptible to being infected with dengue virus
S_v_: Mosquitoes that are susceptible with dengue virus
WHO: World Health Organization
